# Easy or difficult? Investigating perceived ease of changing eating and physical activity behaviors

**DOI:** 10.1111/aphw.70124

**Published:** 2026-02-23

**Authors:** Anila Allmeta, Danielle Arigo, Laura M. König

**Affiliations:** ^1^ Faculty of Life Sciences: Food, Nutrition and Health University of Bayreuth Germany; ^2^ Department Health and Prevention, Institute of Psychology University of Greifswald Germany; ^3^ Department of Psychology Rowan University New Jersey USA; ^4^ Faculty of Psychology University of Vienna Austria

**Keywords:** behavior change, goals, health interventions, self‐efficacy, tailoring

## Abstract

Many people aim to eat healthier or become more physically active, yet often fail. Identifying aspects of behavior that are easier to change is crucial for effective interventions. Two preregistered online studies assessed participants' perceived ease of changing eating and physical activity (PA) behavior and explored potential moderators. Young adults predominantly without (Study 1, N = 435, M_age_ = 31.6) and older adults predominantly with chronic conditions (Study 2, N = 637, M_age_ = 57.2) indicated the perceived ease of changing 21 aspects of eating and PA, medical history, social comparison, prior behavior change attempts and current behavior. Young adults found increasing consumption and engaging in high‐intensity PA easiest and eating less and spending less time sitting most difficult to change. Older adults found reducing consumption and sedentary behavior easiest, and increasing consumption and walking at least 10.000 steps most difficult to change. Lower unhealthy food consumption correlated with easier reduction, while high PA did not always translate to perceiving further increases in PA easier. Results regarding prior attempts and social comparison were mixed across behaviors and samples. Tailored intervention design should integrate users' perceived easiness to change relevant aspects of the target behavior, which may change based on age.

## INTRODUCTION

Many people aim to eat healthier (Ruf & König, 2025) or become more physically active (Oscarsson et al., 2020), yet attempts to change behavior often fail (Conner & Sparks, 2005). Behavior change theories help to understand and facilitate lifestyle modifications, including eating and physical activity (PA). The Health Action Process Approach (HAPA; Schwarzer, 2008), for example, postulates that action and coping planning facilitate putting intentions into action by specifying and linking characteristics of a situation (when and where) and a concrete action sequence (how); the more specific, the more effective these plans should be (Schwarzer, 2008). Goal Setting Theory (GST) (Latham & Locke, 1991) also emphasizes the importance of establishing clear, measurable targets to enhance motivation and commitment towards desired behaviors.

According to GST, to be most effective, goals should be challenging yet achievable (Latham & Locke, 1991). Indeed, it could be hypothesized that some aspects of eating behavior and PA might be easier to change than others, e.g., because they require fewer cognitive resources or time and are easier to integrate into existing routines (Cheval & Boisgontier, 2021; König et al., 2022). For instance, it might be easier to increase the number of steps by getting off the bus one stop early than to make time to schedule a workout session. Steps and activity counts may be more easily modifiable than moderate to vigorous PA since they require less effort and are easier to integrate in existing daily life activities (Cheval & Boisgontier, 2021). Also, it might be easier to introduce healthy eating routines, i.e., eating an apple during the coffee break, or adding a side of vegetables to a lunch instead of just stating the intention to eat healthier overall (Fogg, 2011). This makes it easier to integrate the changes into daily routines as they require less time, money, cognitive, and physical resources to implement, and consequently, perceived ability increases (König & Renner, 2019).

Similarly, Fogg (2009), in his Fogg Behavior Model, emphasizes that the likelihood of successful behavior change can be increased by reducing its complexity and difficulty. He argues that behavior change is more likely to occur if behavior is, for instance, broken down into “baby steps” (i.e., by dividing a demanding behavior such as healthy eating into smaller actions that are more easily accomplished; c.f. “tiny habits”) to increase perceived motivation and ability to achieve the desired change.

Building on these theoretical considerations, it seems prudent to plan behavioral interventions accordingly by either offering a range of goals that most people perceive as attainable, or by steering intervention users towards behaviors that they feel they can actually reach. For this, it is useful to know the perceived difficulty of different aspects of the target behavior; critically, however, information on this is currently lacking. To address this gap, we present results from two studies that systematically assessed participants' perception of the ease of changing different aspects of eating behavior and PA. Kaushal and Rhodes (2015) operationalized behavior complexity as participants' perceived difficulty (and self‐efficacy) in engaging in the behavior, including how hard it is to do, how much effort it takes, and whether they have the physical ability to do the behavior. In line with this, we conceptualized “ease” as individuals' composite perception, which may be rooted in psychological factors including self‐efficacy (Bandura, 1997), cognitive load (Ajzen, 1991), or contextual factors such as access to facilities.

Since it can be assumed that behaviors are perceived to be easier to change if participants already engage in them to some extent (Ouellette & Wood, 1998), we also took previous behavior change attempts and habitual eating behavior and PA into account. Additionally, we present the relationship with medical history, since chronic conditions often require individuals to modify their self‐perceptions and, depending on the condition, limit or eliminate usual functions because of the symptoms or consequences of treatment (Arigo et al., 2014).

Finally, we examined the role of social comparison, or evaluating one's standing relative to others (Festinger, 1954). Comparison plays a key role in forming self‐evaluations of behavioral abilities and performance (Collins, 1996; Dakin & Arrowood, 1981; Ruble et al., 1980) and has important implications for well‐being and health (Gerber et al., 2018). This has been demonstrated with respect to physical health outcomes such as weight loss (Leahey et al., 2011) and in chronic illness self‐care (Arigo et al., 2014). Comparison is also a core mechanism of action in health behavior change interventions such as those to improve physical activity and eating behavior (Olander et al., 2013). Interventions often prompt comparisons between participants to provide information about one's relative performance with respect to the target health behaviors; this is expected to trigger re‐evaluation of one's performance and bolster motivation for increasing healthy behaviors (Arigo et al., 2020). For example, when offered the opportunity to select a peer profile to read, insufficiently active women who selected “highly active” peers were often surprised that these peers' activity was similar to their own; they described re‐evaluating their own activity to be better than their prior self‐perceptions and a resulting increase in motivation to engage in physical activity, as increasing seemed easier after exposure to a successful peer (Arigo et al., 2025). Given the relevance of comparison for evaluating one's abilities and changing health behaviors in the context of interventions, it is likely that comparisons are related to the perceived ease of changing said behaviors. As this relation has received little attention, in the present study, we explored whether comparisons specific to eating and PA were related to the perceived ease of changing aspects of these behaviors.

This research consists of two preregistered exploratory online studies. Specifically, the goal of Study 1 was to explore potential differences in the perceived ease of change of 21 specific aspects of behavior (13 eating‐related and 8 PA‐related) and potential associations with social comparison, previous behavior change attempts, and medical history in a convenience sample of mainly well‐educated young adults, most of whom did not report chronic conditions. With Study 2, we aimed to replicate and extend the findings by additionally investigating associations between the ease of changing eating behavior and PA with habitual levels of these behaviors in a sample of middle‐aged and older adults with a higher likelihood of chronic conditions.

## METHODS

### Study 1

This study was preregistered on the Open Science Framework (OSF; https://osf.io/pzwb7/), and all study materials and data are available from the OSF (https://osf.io/pukdg/). The study protocol was approved by the ethics committee of the University of Bayreuth.

#### Sample

A power analysis in GPower 3.1 (Faul et al., 2009) indicated a sample size of *N* = 435 for an alpha level of .001 (ɑ‐level reduced due to the large number of pairwise comparisons; Faul et al., 2009) using two‐tailed dependent samples t‐tests and an alpha level of .05 using 2 × 2 within‐subjects ANOVAs, and a small effect size (Cohen's d = 0.2 or partial ƞ^2^ = 0.1) (Cohen, 1992) in direct contrasts, with at least 80% power. Inclusion criteria were being at least 18 years old and having a good command of the English language. Exclusion criteria, given the nature of the survey questions, were adults with active eating disorders or disordered eating symptoms (self‐identified). A total of 435 participants completed the questionnaire; they were recruited through social media and word of mouth in winter 2022. Their mean age was 31.6 years (*SD* = 11.4), with 68.5% women, 30.8% men, and 0.7% indicating another gender. Most participants identified as not Hispanic or Latino (93.6%), and 91.5% identified as White. Most of the participants held a university degree, where 38.9% indicated a Bachelor's degree, and 29.9% indicated a Master's degree as their highest qualification. The mean Body‐Mass Index (BMI; kg/m^2^) of the participants was 23.5 (*SD* = 4.8), and 83% of the sample did not indicate any chronic condition.

#### Study design and procedure

A descriptive exploratory online study was conducted using the survey platform Tivian Unipark. All participants provided informed consent prior to participating. Participants did not receive any compensation or feedback. First, participants provided demographic, anthropometric, and basic medical information. Then they were asked to compare their eating and PA behaviors to an average person similar to their demographic characteristics and living conditions to indicate how often they thought about their eating behavior and PA, as well as to indicate previous attempts to change these behaviors. Finally, the participants were asked to indicate how easy or difficult it would be for them to change a total of 21 specific behaviors related to eating and PA.

#### Materials and measures

##### Demographic, anthropometric, and medical history information

Participants were asked to indicate their age (in years), gender (male/female/other), and their education level (i.e., some high school, to 8th grade, some college, no degree, Bachelor's degree, Master's degree). They were also asked to indicate their ethnic or racial heritage and to specify their race. They also provided anthropometric information, i.e. height (in meters) and weight (in kilograms/pounds), based on which we calculated the BMI (kg/m^2^). Additionally, participants reported basic medical information based on which they were later categorized into *without* (if they reported no chronic conditions) vs *with chronic conditions* (if they reported at least one chronic condition). The list included the following conditions: arthritis, asthma, congestive heart failure, COPD (lung disease), diabetes mellitus, hyperlipidemia (high cholesterol), hypertension (high blood pressure), hypothyroidism, myocardial infarction (heart attack), nerve/muscle disease, osteoporosis, and stroke.

##### Social comparison

Social comparison regarding eating behavior was assessed with 2 items using 5‐point scales ((1) much less healthy to (5) much healthier and (1) never to (5) very frequently). Participants rated, relative to the average person, how healthy they thought their eating behaviors were and how often they thought about their own eating behaviors relative to others'. Social comparison regarding PA was assessed with the same two 2 items referring to PA. These items were based on those used in Arigo et al. (2023) and modified to address the context of the present study.

##### Previous attempts to change eating and PA behaviors

The attempt to change eating behaviors, independent of its success, was assessed with 4 items. Participants were asked to indicate the number of times (in the past year) that they attempted to improve their eating behavior, started a formal eating program, started a healthy eating program on their own that lasted 3 days or less, or that lasted more than 3 days in the past year in an open text field. The attempts to change PA levels were assessed with the same 4 items referring to PA.

##### Perceived ease of changing eating and PA behaviors

**FIGURE 1:**
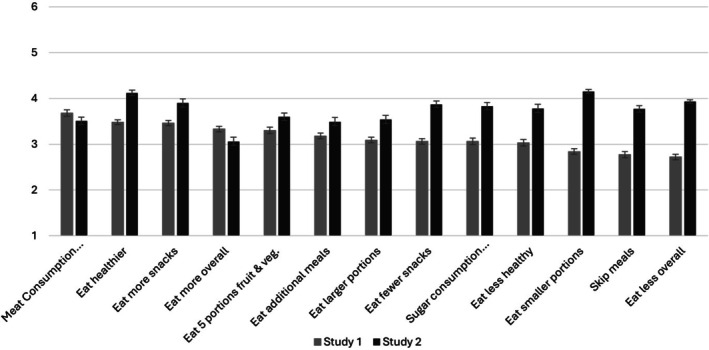
Means and standard errors of the mean of perceived ease of changing eating behaviors, presented separately for the samples in study 1 and study 2.

**FIGURE 2:**
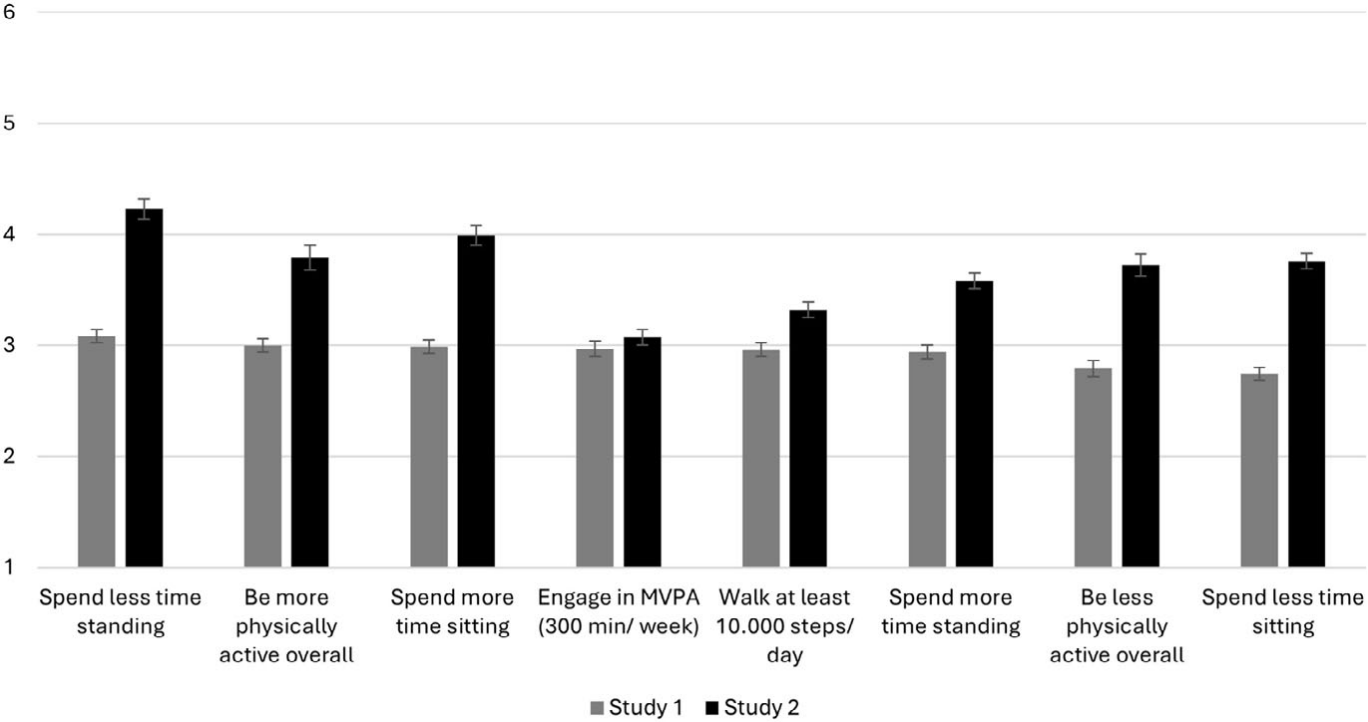
Means and standard errors of the mean of perceived ease of changing physical activity behaviors, presented separately for the samples in study 1 and study 2.

The perceived ease of change of 21 aspects of behavior was assessed (for mean and standard errors of the mean see Figure 1‐ Eating‐related aspects and Figure 2‐ Physical activity aspects; Table A. in supplementary materials for all items means and standard deviations). These included both aspects of behavior that are usually considered “desired changes”, e.g., *eat healthier, eat fewer snacks, be more physically active, spend less time sitting*, as well as aspects that are usually considered “undesired changes”, e.g., *eat less healthy, skip meals, be less physically active, spend more time sitting*, to be able to paint a full picture of behavioral changes in both “healthy” and “unhealthy” directions. Additionally, 5 out of the 21 aspects of behavior (“*eating 5 portions of fruits and vegetables per day”* (WHO, 2003)*, “eating no more than 300 to 600 grams of meat per week”* (Clinton et al., 2020)*, “eating no more than 50 grams of added sugar per day”* (WHO, 2015), *“walking at least 10.000 steps per day”* (CQ University's Physical Activity Research Group, 2001)*, “engaging in moderate to high intensity activities for at least 300 minutes per week”* (Bull et al., 2020)), were based on international recommendations and guidelines. The terms added sugar and moderate to high intensity activities were further defined in a footnote; see the materials on the OSF.

Participants were asked to rate the 21 aspects on a six‐point scale from (1) would be very difficult for me to (6) would be very easy for me. When rating, they were explicitly asked to compare each behavior listed to what they were currently doing.

#### Statistical analysis

Analyses were conducted using IBM SPSS Statistics software version 30.0.0. All participants completed the study questionnaire and there were no missing values for the outcomes of interest. The 13 aspects of eating behavior and 8 aspects of PA were compared using within‐subject ANOVAs. Exploratory pairwise comparisons between each combination of items were conducted to follow up significant main effects. Pearson correlations between the 21 aspects and social comparison and the number of previous behavior change attempts were conducted. Diverging from the pre‐registration, independent samples t‐tests were conducted to compare perceived ease of changing aspects of eating and PA behaviors between participants with and without chronic conditions. Results were considered statistically significant at ɑ = 0.05 for the omnibus test and at ɑ = 0.001 for the pairwise comparisons (due to the large number of relevant comparisons). Effect sizes, respective to the statistical tests conducted, i.e., Cohen's *d*, Pearson's *r*, were interpreted according to Cohen (1992).

### Study 2

This analysis used data from a larger study on cognitive decline (Jeong et al., 2025). This analysis was preregistered on the Open Science Framework (OSF; https://osf.io/x7dyb/), and all study materials and data relevant to this analysis are available from the OSF (https://osf.io/pukdg/). The study protocol was approved by the Institutional Review Board at Clemson University.

#### Sample

Since the research questions addressed in this study were not the primary aim of this data collection, no formal sample size planning for this study was conducted. However, a power analysis in GPower 3.1 (Faul et al., 2009) confirmed that the target sample size of *N* = 400^1^ for the larger project would be sufficient to detect small effects (Cohen's d = 0.2 and partial ƞ^2^ = 0.1) in direct contrasts using two‐tailed dependent samples t‐tests for an alpha level of .001 (ɑ‐level reduced due to the large number of pairwise comparisons) (Faul et al., 2009) and in contrast using 2 × 2 within subjects ANOVAs; with at least 80% power. Inclusion criteria were being at least 40 years old.^2^ having a good command of the English language, living independently, and not being diagnosed with dementia or other cognitive impairment.

A total of 658 participants completed the questionnaire; they were recruited online via the Qualtrics panel in the USA in spring and summer 2023. In the final data analysis, 637 participants were included; 19 participants were removed since they indicated they were younger than 40 and 2 were removed because they did not answer at least 25% of the questions. Participants' mean age was 57.2 years (*SD* = 12.6), with 49.5% women, 49.8% men, and 0.7% indicating another gender. Most of the participants (94%) identified as not Hispanic/Latino/Spanish and the majority (79.6%) identified as White; 22.8% held a Bachelor's degree, and 12.6% a Master's degree. The majority of participants (82.4%) indicated having at least one of the chronic medical conditions listed in the questionnaire.

#### Study design and procedure

Data were collected online as part of a larger study on cognitive decline and social comparison in older adults conducted via Qualtrics. All participants provided informed consent prior to participating in the study. Participants were compensated based on the agreement they completed when joining the Qualtrics survey panel. For example, some participants were compensated with travel rewards (e.g., airline miles) if they joined Qualtrics through their travel rewards program.

Participants provided demographic, anthropometric, self‐rated health, and medical history information. Afterwards, participants completed questionnaires on personality traits, mental health, life satisfaction, cognition, and coping. Then they were asked to compare their eating and PA behaviors to the average person similar to their demographic characteristics and living conditions, to indicate how often they thought about their eating behavior and PA, as well as to indicate previous attempts to change these behaviors. Consequently, the participants were asked to indicate how easy or difficult it would be for them to change a total of 21 specific behaviors related to eating and PA. Finally, they were asked to complete the short version of the International Physical Activity Questionnaire (IPAQ) (Craig et al., 2003) and a Food Frequency Questionnaire (FFQ) (Winkler & Döring, 1998).

#### Materials and measures

Demographic, anthropometric, and medical history information, social comparison regarding eating behavior and PA, previous attempts to change eating behavior and PA, and the perceived ease of changing 21 aspects of eating behavior and PA were assessed in the same manner as in Study 1.

##### Eating behavior

Participants were asked to complete the FFQ by Winkler and Döring (1998), which assesses consumption frequency of 24 food groups on a scale from 1 = almost every day to 6 = never. Based on the scoring algorithm proposed by Winkler and Döring (1998), an overall evaluation score of participants' eating behavior was calculated. In addition, for individual food groups related to individual aspects of behaviors (see Table 1), variables were grouped to represent consumption frequency in line with the behavioral aspect of interest.

**TABLE 1:** Correspondence between: FFQ items and aspects of eating behavior. Summary of correlation analyses conducted in study 2.

FFQ	Aspects of eating behavior	r	df	*p*‐value
Overall score based on Winkler and Döring (1995)	Eating healthier Eating less healthy	.26	509	<.001*
		−.19	508	<.001*
Chocolate, pralines Cakes, pastries, biscuits Other sweets (candys, compote) Fruit juices, other soft drinks (lemonade, cola‐beverages, and others) Diet lemonades, other diet beverages	Eating no more than 50 g of added sugar per day	.11	505	.017*
		.15	503	<.001*
		.20	504	<.001*
		.05	504	.227
		−.07	503	.105
Meat (without sausages) Sausages, ham Poultry	Eating no more than 300 to 600 g of meat per week	.28	501	<.001*
		.16	500	<.001*
		.06	501	.164
Salad or vegetable, raw Vegetable, cooked Fresh fruit	Eating five portions of fruits and vegetables per day	−.38	506	<.001*
		−.33	507	<.001*
		−.41	507	<.001*
Salted snacks, such as salted peanuts, crisps, etc. Chocolate, pralines Cakes, pastries, biscuits	Eating fewer snacks	.22	508	<.001*
		.14	509	.002*
		.11	507	.010*
Other sweets (candys, compote)		.10	508	.031*
Salted snacks, such as salted peanuts, crisps, etc.	Eating more snacks	−.17	508	<.001*
Chocolate, pralines		−.15	509	<.001*
Cakes, pastries, biscuits		−.16	507	<.001*
Other sweets (candys, compote)		−.21	507	<.001*

Abbreviation: FFQ = Food Frequency Questionnaire.

* Correlations significant at .005 level (2‐tailed).

##### Physical activity

PA was assessed by asking the participants to complete the short version of the IPAQ (IPAQ‐SF). Due to an error in the set‐up of the survey, participants reported the activity time in ranges and not hours and minutes. Therefore, diverging from the preregistration, instead of considering the collected data as continuous variables (calculating MET‐minutes), the data were treated as categorical and further processed following the scoring algorithm of the IPAQ‐SF (Sjostrom 2005). Participants were categorized into three levels of PA: low, moderate, and high. Walking and sitting time were considered as ordinal variables. The corresponding IPAQ scores and items and the aspects of PA assessed in the questionnaire are presented in Table 2.

**TABLE 2:** Correspondence between: IPAQ‐SF scores and aspects of physical activity. Summary of analyses conducted in study 2.

IPAQ	Aspects of physical activity	t	df	p‐value	Cohen's *d*	Yes		No	
						M	SD	M	SD
Low physical activity	Being more physically active	3.61	508	.495	1.50	3.62	1.49	4.28	1.59
	Being less physically active	−5.27	501	.279	1.51	3.96	1.49	2.97	1.63
Moderate physical activity	Being more physically active	−3.69	508	365	1.50	4.30	1.60	3.61	1.48
	Being less physically active	4.80	501	.161	1.52	3.04	1.67	3.94	1.49
High physical activity	Being more physically active	−7.86	508	.026*	1.44	4.45	1.33	3.38	1.48
	Being less physically active	4.96	501	.001*	1.52	3.31	1.65	4.03	1.45
Moderate physical level	Engage in moderate to high intensity activities for at least 300 min/week	−7.29	507	.659	1.60	4.30	1.56	2.86	1.60
High physical activity level		−12.35	507	.446	1.47	4.26	1.53	2.53	1.44
		**Spearman’s r*ho***				***df***		***p*‐value**	
Walking time	Walking at least 10,000 steps/day	.41				505		<.001*	
Sitting time	Spending less time sitting	−.27				498		<.001*	
	Spending more time sitting	.20				500		<.001*	

Abbreviation: IPAQ‐SF = International Physical Activity Questionnaire – Short Form.

* significant at .005 level (2‐tailed).

#### Statistical analysis

Analyses were conducted using IBM SPSS Statistic software version 30.0.0.0. Missing values were deleted case‐wise. Missing values did not exceed 20% of the total number of participants (18.4%–19.3%). Nevertheless, in each statistical test, the number of participants was never below the sample size (*n* = 400) calculated to achieve at least 80% power and detect a small effect size (Cohen's d = 0.2). In line with Study 1 but diverging from the preregistration, the 13 aspects of eating behavior and 8 aspects of PA were compared using within‐subject ANOVAs. Exploratory pairwise comparisons between each combination of items were conducted to follow up on significant main effects. Additionally, to align with the analysis of Study 1, Pearson correlations between the 21 aspects and social comparison and the number of previous behavior change attempts were conducted. The associations between perceived ease of change and the frequency of consuming certain foods and beverages, as indicated in the FFQ, were analyzed with Pearson correlations. Diverging from the preregistration but in line with the adjusted coding of the IPAQ, the associations between perceived ease of change and the IPAQ scores and items were tested using independent samples t‐tests (for categorical variables) and Spearman rank correlations (for ordinal variables). Lastly, the association between perceived ease of change and medical history (with vs without chronic conditions) was tested using independent samples t‐tests. Results were considered statistically significant at ɑ = 0.05 for the omnibus test and at ɑ = 0.001 for the pairwise comparisons (due to the large number of relevant comparisons). Results were interpreted based on the effect size, respective to the statistical tests conducted, i.e., Cohen's *d*; Pearson's *r*, according to Cohen (1992).

## RESULTS

### Study 1

#### Do aspects of eating behavior and PA differ in the perceived ease of change?

A within‐subjects ANOVA yielded significant differences for aspects of eating behavior (*F*[12, 423] = 21.08, *p* < .001, partial ƞ^2^ = 0.37). Participants perceived “*eat[ing] no more than 300‐600 grams of meat per week”* to be the easiest aspect to change (*M* = 3.68, *SD* = 1.54) and “*eat[ing] less overall*” to be the most difficult (*M* = 2.72, *SD* = 1.20). In Table A in the supplementary material, means and standard deviations for each of the 13 aspects of eating behavior are reported; results of all post hoc paired comparisons are listed in the supplementary material, Table B. In brief, there were significant differences for almost all pairwise comparisons between the 13 aspects of eating behavior (*p*‐values .049 to <.001). Aspects of eating behavior where individuals would increase consumption, i.e., eat more overall, eat more snacks, eat healthier, or eat 5 portions of fruit and vegetables per day, were generally perceived as easier to change in comparison to aspects where they would need to reduce the consumption, i.e., eat less overall, eat smaller portions, or skip meals.

Significant differences were also found for aspects of PA (*F*[7, 428] = 5.28, *p* < .001, partial ƞ^2^ = 0.08). Participants perceived “*spending less time standing*” (M = 3.08, *SD* = 1.22) to be the easiest aspect to change and “*spending less time sitting*” (M = 2.74, *SD* = 1.25) to be the most difficult aspect to change. In Table A (in the supplementary material), means and standard deviations for each of the 8 aspects of PA are reported; post‐hoc paired comparisons are listed in the supplementary material, Table C. In brief, aspects such as being more physically active, engaging in moderate to vigorous physical activity for 300 min/week, walking at least 10.000 steps/day seem to be perceived as easier to change in comparison to aspects such as spending less time sitting, spending more time standing, or being less physically active overall. Mainly, there were significant differences only between the following aspects of PA: be more physically active, spend less time sitting, and engage in moderate‐vigorous physical activity for at least 300 min/week, and walk at least 10.000 steps/day; *p‐*values ranged from .048 to <.001.

#### Are social comparisons associated with perceived ease of change?

Pearson correlations were conducted to examine the relationship between social comparison and the perceived ease of changing eating and PA behaviors. All results are presented in the supplementary material, Table D. In brief, small to medium significant positive correlations were found between participants' ratings on the healthiness of their eating behavior in comparison to the average person with similar characteristics as themselves and the ease of changing healthy behaviors such as “*eat[ing] smaller portions*” (r = .10, *p* = .034), “*eat[ing] healthier overall*” (r = .39, *p* < .001), or “*eat[ing] fewer snacks*” (r = .20, *p* < .001), indicating that people who think they eat healthier than others also find it easier to engage in these behaviors. There was also observed a negative significant correlation with the item “*eat less healthy* (r = ‐.33, *p* = <.001)”, indicating that participants who think that they eat healthier than others found it more difficult to eat less healthy. In addition, there were small significant positive correlations between how often participants thought of their eating behaviors in relative to others and the items “*eat more in general*” (r = .10, *p* = .036) and “*eat larger portions*” (r = .12, *p* = .012), indicating that more frequent social comparisons were associated with participants thinking that eating more and larger portions would be easier for them.

Regarding PA, there was a small but significant positive correlation between participants' rating of the level of their PA in comparison to the average person with similar characteristics as themselves and the item “*engage on moderate/vigorous physical activity for 300min/week*” (r = .13 *p* = .008), indicating that participants who think that they were more physically active than others found it easier to engage in recommended levels of moderate to vigorous PA. Small to medium positive significant correlations were also found between how often participants thought of their PA levels relative to others and desired aspects of PA, including “*be more physical active overall*” (r = .34 *p* < .001)*; “walk 10.000 steps/day*” (r = .34, *p* < .001) and “*engage in moderate/vigorous physical activity for 300min/week*” (r = .60, *p* < .001). In addition, the results showed small to medium negative significant correlations for undesired PA aspects: “*be less physically active overall*” (r = −.37, *p* < .001); “*spend less time standing*” (r = − .14, *p* = .003) and “*spend more time sitting*” (r = −.26, *p* < .001). Taken together, these results indicate that participants who compared themselves more frequently to others regarding their PA behaviors found it easier to be more physically active. All results are presented in the supplementary material, Table E.

#### Are previous behavior change attempts associated with perceived ease of change?

To investigate the associations between previous attempts to change eating behavior and PA with the participants' perceived ease of changing these behaviors, Pearson correlations were conducted (see the supplementary material Tables F and G, respectively, for all results). Small yet significant positive correlations between previous attempts to improve eating behavior and “*eat more overall*” (r = .15, *p* = .002); “*eat larger portions*” (r = .11, *p* = .021); and “*eat less healthy*” (r = .13, *p* = .008) were found, indicating that participants with more prior eating behavior change attempts found it easier to engage in undesired eating behaviors. Also, a small significant positive correlation resulted between the number of times participants started a formal eating program and “*eat additional meals*”. Meanwhile, there were medium to large significant positive correlations between the number of times participants started a formal exercise program and “*walk[ing] 10.000 steps/day*” (r = .10, *p* = .049) and between previous attempts to improve PA level and “*being less physically active*” (r = .11, *p* = .036).

#### Is the medical history associated with perceived ease of change?

Independent samples t‐tests were conducted to compare each of the 13 aspects of eating behavior between participants with and without chronic conditions. The t‐tests showed significant differences between the two groups for four of the 13 eating‐related aspects (see Table 3 for all results). Generally, participants without chronic conditions perceived it to be easier to “*skip meals*” and more difficult to change the other three aspects of eating behavior compared to participants with chronic conditions. In addition, the results of the t‐test showed significant differences between the two groups for three of the eight PA aspects (see Table 4 for all results). Generally, participants without chronic conditions perceived it to be easier to change their PA in comparison to participants with chronic conditions.

**TABLE 3:** Associations between medical history and perceived ease of change in study 1.

Aspects of behavior	t	df	*p*‐value	Cohen's d	Without chronic conditions		With chronic conditions	
					M	SD	M	SD
**Eating behavior:**								
Eat less overall	.03	433	.979	1.20	2.72	1.19	2.72	1.29
Eat smaller portions	−.31	433	.760	1.21	2.83	1.18	2.88	1.35
Eat more overall	.90	433	.367	1.93	3.35	1.27	3.20	1.39
Eat larger portions	.25	433	.802	1.34	3.10	1.32	3.05	1.46
Eat healthier	.53	433	.598	1.10	3.49	1.10	3.42	1.08
Eat less healthy	−.30	433	.767	1.35	3.02	1.35	3.07	1.34
Skip meals	−4.04	433	<.001*	1.40	2.65	1.40	3.36	1.37
Eat additional meals	1.75	433	.081	1.33	3.23	1.31	2.93	1.43
Eat fewer snacks	1.78	433	.076	1.26	3.01	1.25	3.30	1.29
Eat more snacks	2.82	433	.005*	1.22	3.53	1.19	3.09	1.37
Eat 5 portions of fruit and vegetables	2.06	433	.040*	1.45	3.37	1.44	2.99	1.55
Eat no more than 300–600 g of meat/week	2.21	433	.027*	1.53	3.76	1.52	3.32	1.56
Eat no more than 50 g of added sugar/day	1.18	433	.237	1.36	3.10	1.35	2.89	1.40
**Physical activity:**								
Be more physically active overall	2.45	433	.015*	1.18	3.06	1.18	2.69	1.19
Be less physically active overall	.23	433	.816	1.19	2.80	1.38	2.76	1.39
Spend more time standing	1.07	433	.285	1.18	2.97	1.15	2.81	1.33
Spend less time standing	−.35	433	.724	1.22	3.07	1.19	3.12	1.37
Spend less time sitting	.89	433	.371	1.52	2.76	1.25	2.62	1.27
Spend more time sitting	−1.15	433	.252	1.28	2.96	1.26	3.15	1.36
Walk at least 10.000 steps/day	2.29	433	.023*	1.33	3.02	1.31	2.64	1.38
Engage in moderate/vigorous physical activity for 300 min/week	2.73	433	.007*	1.34	3.05	1.32	2.58	1.41

*Note*: *significant at .005 level (2‐tailed).

**TABLE 4:** Associations between medical history and perceived ease of change in study 2.

Aspects of behavior	t	df	*p*‐value	Cohen's d	Without chronic condition		With chronic condition	
					M	SD	M	SD
**Eating behavior:**								
Eat less overall	−2.59	505	.010*	1.32	4.29	1.16	3.85	1.34
Eat smaller portions	−2.33	505	.020*	1.28	4.48	1.17	4.10	1.30
Eat more overall	−.62	502	.536	1.48	3.88	1.37	3.76	1.50
Eat larger portions	−1.37	504	.170	1.53	3.78	1.39	3.52	1.55
Eat healthier	−3.04	505	.003*	1.35	4.55	1.14	4.03	1.39
Eat less healthy	.37	505	.709	1.46	3.74	1.44	3.81	1.46
Skip meals	−.83	506	.406	1.55	3.92	1.61	3.75	1.55
Eat additional meals	−2.47	504	.081	1.33	3.84	1.54	3.44	1.50
Eat fewer snacks	−2.33	505	.020*	1.42	4.26	1.32	3.82	1.44
Eat more snacks	−.86	505	.391	1.46	4.03	1.33	3.87	1.48
Eat 5 portions of fruit and vegetables	−2.92	502	.004*	1.57	4.10	1.57	3.51	1.57
Eat no more than 300–600 g of meat/week	−2.37	497	.018*	1.57	3.92	1.45	3.44	1.59
Eat no more than 50 g of added sugar/day	−1.18	500	.240	1.50	4.01	1.42	3.79	1.51
**Physical activity:**								
Be more physically active overall	−4.19	505	<.011*	1.49	4.38	1.31	3.59	1.52
Be less physically active overall	.74	498	.460	1.55	4.38	1.31	3.59	1.52
Spend more time standing	−5.75	501	<.001*	1.56	4.54	1.27	3.40	1.60
Spend less time standing	1.15	502	.251	1.33	4.28	1.32	4.54	1.27
Spend less time sitting	−3.33	501	<.001*	1.41	3.05	1.32	2.58	1.41
Spend more time sitting	.03	502	.979	1.41	4.01	1.40	4.02	1.41
Walk at least 10.000 steps/day	−7.40	503	<.001*	1.56	4.57	1.15	3.10	1.61
Engage in moderate/vigorous physical activity for 300 min/week	−5.86	503	<.001*	1.62	4.11	1.55	2.90	1.64

*Note*: *significant at .005 level (2‐tailed).

### Study 2

#### Do aspects of eating behavior and PA differ in the perceived ease of change?

A within‐subjects ANOVA yielded significant differences for aspects of eating behavior (*F*(12, 5,868) = 12.38, *p* < .001, partial ƞ^2^ = .025; see Table A in the supplementary material). More specifically, participants perceived “*eat[ing] smaller portions”* to be easiest to change (*M* = 4.15, *SD* = 1.29) and “*eat[ing] additional meals*” to be most difficult to change (*M* = 3.49, *SD* = 1.51). In general, desirable aspects of eating behavior such as eating smaller portions, eating healthier, or eating less overall were perceived as easier to change in comparison to undesirable aspects such as eating additional meals or eating larger portions. Post‐hoc tests revealed differences in almost all pairwise comparisons between the 13 aspects of eating related behavior (*p*‐values .040 to <.001; see supplementary material, Table H).

Significant differences were also found for aspects of PA (*F*(7, 3,465) = 34.67, *p* < .001, partial ƞ^2^ = .065; see Table A in the supplementary material). Participants perceived “*spending less time standing*” (M = 4.23, *SD* = 1.34) to be the easiest to change and “*engage[ing] in at least 300 minutes of moderate to vigorous physical activity per week*” (M = 3.07, *SD* = 1.68) to be the most difficult. In brief, general aspects of behavior such as being overall more physically active, spending less time standing or more time sitting were perceived to be easier to change in comparison to very specific aspects of behavior such as engaging in moderate‐vigorous physical activity for 300 min/week or walking at least 10.000 steps/day. Post‐hoc tests revealed significant differences in almost all paired comparisons (p‐values between .014 and <.001; see supplementary material, Table I).

#### Are social comparisons associated with perceived ease of change?

Although not planned in the pre‐registration, we aligned Study 2 with Study 1 and also investigated whether social comparisons were associated with perceived ease of change. All Pearson correlations and specific descriptions are presented in the supplementary material (Tables J and K for PA and eating behavior, respectively). In brief, small to medium significant positive correlations were found between participants' ratings of the healthiness of their eating behavior in comparison to the average person with similar characteristics as themselves and the ease of changing healthy behaviors such as eating less overall, eating healthier overall, or eating five portions of fruit and vegetables, indicating that participants who think that they eat healthier than others also find it easier to engage in these behaviors. However, there were no significant correlations between how often participants thought of their eating behaviors in relation to others. Regarding PA, participants who compared their PA levels more frequently to others perceived it easier to be more physically active.

#### Are previous behavior change attempts associated with perceived ease of change?

Again, to align Study 2 with Study 1, we also investigated whether previous behavior change attempts were associated with perceived ease of change. All results of the analysis (Pearson's correlations) and specific descriptions are presented in the supplementary material (Tables L and M for eating and PA behavior, respectively). Generally, the results indicate that participants with more prior eating behavior change attempts found it easier to engage in desired eating behaviors. Meanwhile, regarding previous attempts to change PA levels, the results would suggest that participants that started formal exercise programs would perceive it to be more difficult to “*be less physically active*” and easier to “*engage[ing] in mod‐vigorous physical activity for at least 300 min/week*”.

#### Is the medical history associated with perceived ease of change?

Independent samples t‐tests were conducted to compare each of the 13 aspects of eating behavior between participants with and without chronic conditions. The t‐tests showed significant differences between the two groups for seven of the 13 eating‐related aspects (see Table 4 for all results). Generally, participants with chronic conditions perceived it to be more difficult to change these aspects of eating behavior than participants without chronic conditions. In addition, the results of the t‐test showed significant differences between the two groups for five of the eight PA aspects (see Table 4 for all results). Generally, participants with chronic conditions perceive it to be more difficult to change these aspects of PA in comparison to participants without chronic conditions.

#### Is the frequency of consuming specific foods and beverages related to perceived ease of changing related aspects of eating behavior?

To examine the association between the frequency of consuming different foods and beverages, indicated on a scale from (1) nearly every day to (6) never, and the perceived ease of change of the corresponding eating‐related aspects, Pearson's correlations were conducted. Results are summarized in Table 1. Participants who reported lower consumption frequency (represented by higher values on the item) also reported to be easier to change “eat[ing] healthier” and difficult to change “eat[ing] less healthy”.

There were small significant positive correlations between the perceived ease of “*eat[ing] no more than 50g of added sugar per day*” and the consumption frequency of the following food items: *“chocolate, pralines”* “*cakes, pastries, biscuits*” and “*other sweets, candy, compote*”; due to the scoring of the FFQ items, this indicates that higher perceived ease of reducing sugar consumption was associated with lower consumption frequency of unhealthy foods.

Additionally, there were small positive correlations between the perceived ease of change of the meat consumption eating aspect: “*eat no more than 300‐600g of meat/week”* and the consumption of the following food items: “*meat (without sausage*”) and “*sausage, ham*”; due to the scoring of the FFQ items, this indicates that higher perceived ease of reducing meat consumption was associated with lower consumption frequency of meat‐based foods.

Moreover, the correlation analysis revealed moderate, negative correlations between the perceived ease to change “*eat 5 portions of fruit and vegetables per day*” and the following food items: “*salad or vegetables (raw)*” “*vegetables cooked*” and *“fresh fruits;”* due to the scoring of the FFQ items, this indicates that higher perceived ease of increasing fruit and vegetable consumption was associated with higher consumption frequency of fruit and vegetable based foods.

Lastly, there were small, positive correlations between the perceived ease of change regarding snack consumption (“*eat fewer snacks*”) and the consumption frequency of the following food items: “*salted snacks*”; “*chocolates, pralines*”; “*cakes, pastries, biscuits*”; and “*other sweets, candy, compote*”. While there were small, negative correlations between “*eat more snacks*” and “*salted snacks*”; “*chocolates, pralines*”; “*cakes, pastries, biscuits*”; and “*other sweets, candy, compote*”; due to the scoring of the FFQ items, this indicates that higher perceived ease of reducing snack consumption was associated with lower consumption frequency of different types of snacks.

#### Are PA levels associated with the perceived ease of change?

First, we examined the relationship between PA levels and the perceived ease of change of the corresponding PA‐related aspects using independent samples t‐tests. All results are summarized in Table 2. There were no significant differences in the perceived ease of change of “*engage[ing] in moderate‐high intensity physical activity for at least 300 min/week”* and participants that had (or not) a moderate level or the ones that had (or not) a high level of PA.

Regarding the general PA level, independent‐samples t‐tests between each PA category (low, moderate, and high PA level) and the perceived ease of “*being more and being less physically active*” were conducted. There were no significant differences in the perceived ease of change of “*being more physically active*” or “*less physically active*” and the participants that had (not) a low or a moderate level of PA. Instead, there were significant differences in the perceived ease of change of “*being more physically active*” or “*being less physically active*” and the participants that had (or not) a high level of PA: participants that had a higher level of PA would perceive it more difficult to further increase their PA level (“*be more physically active*”) and easier to decrease their PA level (“*be less physically active*”).

Correlations were conducted for the remaining indicators for PA; since the scales were ordinal, Spearman correlations were used. Results are summarized in Table 2. There was a moderate positive correlation between the time spent walking and the perceived ease of “*walking at least 10.000 steps/day*”, thus, participants that spent more time walking, perceived it to be easier to walk at least 10,000 steps a day. Furthermore, there was a small negative correlation between the sitting time and the perceived ease of “*spending less time sitting*” and a small positive correlation and the perceived ease of “*spending more time sitting*”. Thus, participants who spent more time sitting perceived it to be more difficult to reduce their sitting time, and instead perceived it easier to increase the time they spent sitting.

## DISCUSSION

Health behavior theories suggest that achievable goals facilitate behavior change. This research thus was designed to assess participants' perceptions of the ease of changing a broad range of eating and physical activity behaviors to guide tailored interventions. We also explored how age, medical history, previous attempts to change behavior, social comparisons, and frequency of consumption of specific foods and beverages, physical activity, and sitting time are associated with these perceptions. Overall, different aspects of eating and PA were indeed seen as differing in ease to change. These differences were related to age and medical history, suggesting a potential influence of an individual's physical health status and associated external factors. Furthermore, at least for eating behaviors, healthier food intake was associated with finding engaging in further healthy eating behaviors easier; this is further supported by the finding that prior behavior change attempts were also associated with perceived ease. Together, these results underline the complexity, but also the necessity, of tailoring interventions to individual needs to promote their effectiveness.

Overall, the observation that the participants in this study indeed viewed different aspects of eating and PA as differing in ease to change mirrors prior research. Using a similar method to our study, Dorina et al. (2024) asked participants to indicate the complexity of 24 behaviors related to health and sustainability, showing that perceived complexity is influenced by several salient characteristics and that complex behaviors (e.g., abstaining from smoking) are more likely to be true to the previously identified characteristics compared to simple behaviors (e.g., eating fruit). Importantly, however, Dorina et al. (2024) asked participants to rate the complexity for an average person, while the present research focused on individuals' perceptions for themselves. Additionally, they focused on different behaviors, while in this study the focus is on different aspects of two behaviors (eating‐related and PA behavior). The perceived difficulty or ease of changing a behavior is likely rooted in a complex interplay of psychological factors. According to Kaushal and Rhodes (2015), the perceived difficulty of a behavior is influenced by self‐efficacy, with McCloskey and Johnson (2019) additionally suggesting that the time, attention, and planning required to prepare and execute a behavior are also considered. Attempting to change perceived ease of behavior thus likely requires a multifaceted intervention, providing further support for the underlying assumption of the Fogg Behavior Model (Fogg, 2009) to adjust the intervention's target behavior instead to increase motivation.

### Differences based on age and medical history

Regarding eating behavior, younger adults predominantly without chronic conditions (Study 1 sample) found increases in consumption easier, which might align with hedonic goals and social facilitation of eating, that emphasize pleasure, satiety, and shared experiences over strict dietary control (McInnes et al., 2023; Stok et al., 2016; Vartanian et al., 2007). Without immediate need for dietary restrictions, the notion of “*eating more*” can feel less effortful than “*eating less”*, which would involve restraint and perceived deprivation. Anticipated changes might thus be viewed through the lens of convenience, taste, or social acceptance rather than broad health management as could be the case for adults with chronic conditions (McInnes et al., 2023). In contrast, participants predominantly with chronic conditions (Study 2 sample) found it easier to make healthier choices i.e., *eating smaller portions* in comparison to less healthy choices i.e., *eating additional meals*, which could be driven by higher health motivation (c.f. extrinsic adaptive motivation; Ryan & Deci, 2017; Ahmadi et al., 2023) and clearer outcome expectancies directly linked to their health status (Rosenstock et al., 1988). For individuals managing chronic conditions, the link between dietary choices and disease management becomes notable and often a primary motivator. They may have developed self‐efficacy through past experiences or guidance from healthcare professionals, recognizing that these changes, while requiring effort, yield considerable health benefits that are highly valued (Bandura, 1997). The awareness of the importance of healthy choices, not only for general health but also for preventing or coping with specific conditions, might reorient their perception of “ease”. What might be perceived as restrictive by a healthy young adult might become a necessary and therefore more “doable” (or even empowering) strategy for managing their health for someone with a chronic illness.

Both groups perceiving increases in PA (compared to decreases in PA) as easier to change is consistent with an overarching positive societal value placed on being active (Sallis & Owen, 1999). However, young adults (Study 1 sample) favoring concrete increases (i.e., *300 min MVPA/week, 10,000 steps/day*), while adults with chronic conditions (Study 2 sample) preferring *less time sitting* or *more time standing* indicates how perceived capability and different barriers might influence the perception of ease. For instance, for young adults, who face fewer health‐related limitations, specific and challenging PA goals like achieving a certain step count or vigorous activity duration are often perceived as highly attainable (Locke & Latham, 2002). Their self‐efficacy for these performance‐based targets is likely robust, derived from past successes and a general expectation of physical capability. This aligns with goal‐setting theory, which states that specific, difficult goals can be highly motivating—both intrinsically and extrinsically—and lead to greater effort and achievement, especially when individuals believe they have the means to attain them (Locke & Latham, 2002; Swann et al., 2025). Instead, for adults with chronic conditions (Study 2 sample), the perceived ease associated with reducing sedentary behavior rather than increasing vigorous activity could be adaptive. For this population, traditional PA goals might be impacted by symptoms such as pain, fatigue, or functional limitations, making large increases in structured exercise feel daunting and potentially risky (Ahmadi et al., 2023; Rebar et al., 2016; Ryan & Deci, 2017; Shields et al., 2012). Reducing sedentary time, while still challenging due to being strongly habitual, might still be perceived as a more manageable and less physically demanding target (Gardner et al., 2012). This reflects a pragmatic approach to perceived behavioral control, where individuals prioritize behaviors they feel more confident about, given their health context.

Additionally, age and health status might also shift the behavior change approach from specific, tangible actions to more holistic, long‐term lifestyle modifications. For instance, for younger adults and healthy individuals in Study 1, achieving specific, tangible goals derived from guidelines might provide immediate positive reinforcement, making those aspects seem easier and more directly impactful on their daily lives. In contrast, older adults (Study 2), especially those managing complex health conditions, might shift their focus towards maintaining health and well‐being rather than achieving specific, potentially difficult, performance‐oriented goals. At the same time, broad changes like “be[*ing*] more active overall” might feel especially difficult due to the expected increased cognitive load and the cumulative nature of managing multiple health aspects, making small, specific “wins” less impactful or harder to identify within a larger, more ambiguous goal (Charness & Boot, 2016). Sawyer and McManus (2021) qualitatively explored the experience of adults with chronic conditions such as type 2 diabetes and hypertension attempting lifestyle changes including PA and dietary habits. Their results indeed showed that participants often felt overwhelmed by the gap between the current and the desired state, suggesting that broader, more general goals like “losing weight” or “eating healthier” felt daunting without breaking them down into smaller, manageable steps. Therefore, interventions must consider these distinct perceptions, setting realistic and personally relevant goals, whether they are performance‐based for healthy individuals or focus on modifying sedentary routines for those with chronic conditions to maximize perceived ease of change and, consequently, engagement and adherence (Hutchesson et al., 2015; McAuley et al., 2003; Michie et al., 2009; Neupert et al., 2009; Rebar et al., 2016; Schau et al., 2025).

### Associations with eating‐ and activity‐based social comparison

We identified several positive associations between ratings of eating behaviors and PA, respectively, as healthier than that of others and perceived ease of changing related eating and PA behaviors. Indeed, individuals who perceive their current behaviors as superior to those of their peers may develop a stronger sense of self‐efficacy via mastery experiences (Bandura, 1997; Strecher et al., 1986), believing that, since they are already doing well, further improvements are more easily achieved. Furthermore, social comparison theory suggests that downward social comparison can boost self‐esteem and confidence, further reinforcing the belief in one's capability to succeed (Spray et al., 2023; Strecher et al., 1986).

When asked about comparison frequency, associations with perceived ease of changing diverged between the two study populations. For the young adult sample, a higher comparison frequency correlated with increased perceived ease of eating more, which could be influenced by prevailing social norms and hedonic goals within their peer groups (Stok et al., 2016). In many social settings, particularly among young adults, eating larger portions or indulgent foods might be normalized or even encouraged within social gatherings, leading individuals to perceive such behaviors as less effortful or more socially acceptable (Sogari et al., 2018). For older adults, frequent social comparisons were associated with perceived ease of eating less and healthier, which suggests a different comparative framework. In this group, social comparison may involve observing peers or support networks effectively managing their conditions through dietary discipline. Such upward comparisons can provide examples of success and enhance self‐efficacy for one's own health‐promoting behaviors, making more restrictive or healthier eating goals seem more achievable (Arigo et al., 2020; Bandura, 1997; Buunk & Ybema, 1997). This highlights how health status and life stage shape the relevance and interpretation of social comparison information.

### Associations with prior behavior change attempts

Prior behavior change attempts also had divergent associations in the two samples and for the two behaviors of interest. For the young adult sample, prior attempts to change eating behavior were associated with perceiving “undesired/ unhealthy” behaviors to be easier to adopt. This aligns with self‐regulation theories where repeated experiences of success or failure can shape future behavioral predictions (Ouellette & Wood, 1998); in this case, failed attempts may have induced a feeling of restriction being too difficult, or (excessive) consumption being much easier. In contrast, for the older adult sample, prior efforts might lead to a perceived ease of healthy eating, which suggests a different learning trajectory. For this group, previous engagement in behavior change may have provided valuable mastery experiences or increased perceived behavioral control. The seriousness of their health conditions might also enhance their intent to learn from past attempts, viewing any partial success as evidence of their capability to achieve healthier outcomes, thus increasing their self‐efficacy for adaptive changes (Bandura, 1997; Kwasnicka et al., 2016; Sniehotta et al., 2014).

Interestingly, patterns were opposite for PA, where for the young adult sample, engagement in formal exercise programs was associated with greater perceived ease of changing PA, which may indicate that program participation has bolstered their self‐efficacy and confidence. Indeed, structured programs can provide clear guidance, opportunities for skill mastery, and positive feedback, thereby enhancing perceived behavioral control and making specific activity goals seem more attainable (Ryan & Deci, 2017). For the older adult sample, however, participation in formal exercise programs was associated with perceiving becoming more physically active more difficult, suggesting that these programs might highlight the significant barriers and effort involved in managing PA alongside their health challenges (Bauman et al., 2012; Shields et al., 2012). Additionally, when asked to indicate the number of times that they had started a formal program that lasted more or less than 3 days, especially in the older adult sample, the more they indicated to have started such a program (lasting 3 or more days), it was associated with a higher perceived ease to change healthy aspects of eating behavior such as “eat less overall” or “eat no more than 50g added sugar/day”, suggesting that repeated attempts may reflect persistence. Importantly, we only assessed whether participants had previously attempted to change their behavior without directly asking whether they were successful; the interpretation of the diverging patterns thus needs to be confirmed in future research.

### Associations with current behaviors

The observation that lower consumption of sweets and meat‐based foods correlated with a perceived ease in reducing added sugar and overall meat intake, respectively, aligns with the principle that weaker existing habits are generally easier to alter or cease (Gardner et al., 2012) since they demand less cognitive effort to resist or reduce them further. Conversely, a higher frequency of consuming vegetable‐based foods being associated with a perceived ease in meeting a 5‐portions‐a‐day target highlights the reinforcement of positive habit formation. Consistent engagement in a desirable behavior builds self‐efficacy via mastery experiences, making the continued or increased performance of that behavior seem less effortful and more attainable (Bandura, 1997; Ouellette & Wood, 1998).

The lack of association between moderate/high PA levels and the perceived ease of changing to “*engage in moderate‐high PA for at least 300 min/week*” may suggest a ceiling effect for perceived ease especially among already active individuals with chronic conditions. While these participants demonstrate high self‐efficacy for their current activity levels, the target of 300 minutes per week of moderate‐to‐vigorous PA is substantial, especially when managing ongoing health issues. For individuals already meeting a certain threshold, further increases may represent a disproportionate increase in perceived effort or a conflict with other life demands or symptom management, even if they are physically capable. Thus, “more” is not necessarily “easier”, as also the findings for highly active participants suggest. Their high PA level might already be close to their physiological or psychological limits, and any further increase could be perceived as demanding an unsustainable level of effort, risking pain, fatigue, or symptom exacerbation (Shields et al., 2012; Webb et al., 2022). Contrarily, “*being less active overall”* is less demanding and would reduce effort, in line with the concept of effort avoidance in decision‐making around behavior, where less effortful options are often perceived as easier (Bustamante et al., 2024). Moreover, the finding that participants who spent more time sitting perceived it as difficult to “*spend less time sitting*” but easier to “*spend more time sitting*” strongly points to the power of habit strength and behavioral inertia (Gardner et al., 2024, 2012). Sedentary behaviors, such as sitting, are often deeply ingrained in daily routines, frequently influenced by environmental cues (e.g., office work, home entertainment) and requiring little conscious effort to perform (Gardner et al., 2024, 2012). Breaking such established habits or routines demands significant self‐regulation and sustained effort, which may feel overwhelming.

### Avenues for future research

Importantly, the present work was based on the assumptions of established health psychology theories that postulate that setting concrete goals is crucial for behavior change (HAPA‐ Schwarzer, 2008; GST ‐ Latham & Locke, 1991). Another line of work has emphasized the effectiveness of “open goals” (or non‐specific goals) in promoting PA and increasing positive psychological experiences, particularly for individuals who are not currently active (Clarke et al., 2025). Open goals, which involve exploring performance (e.g., “see how far you can walk in 6 minutes”), lead to higher interest in repeating the activity and higher motivation for future engagement compared to SMART goals (specific, measurable, achievable, relevant, time‐bound) for certain groups (Clarke et al., 2025; Swann, 2012; Swann et al., 2020, 2025). While the present research did not include these types of goals, replicating the findings based on the open goals account would be valuable.

Future research should also employ longitudinal designs to establish causality and track how perceptions of ease evolve with actual behavior change attempts and health outcomes. Moreover, larger sample sizes could allow for additional analyses, i.e., regression analysis to test relationships between the perceived ease of change rating and different predictors, also including several covariates. Investigating the underlying cognitive and affective processes (i.e., specific outcome expectancies, emotional responses to effort, habit strength) that drive these differential perceptions through qualitative methods or experimental designs would also be valuable. Furthermore, studies could explore the effectiveness of interventions explicitly tailored to these distinct perceptions of ease across different age and health groups, and examine how digital health technologies can facilitate personalized goal setting and feedback (Krukowski et al., 2024).

### Study limitations

This study, while offering valuable insights, is subject to several limitations. A primary limitation is its cross‐sectional nature, which precludes the testing of causal relations (Levin, 2006); the associations present in this work have to be interpreted with caution and directionality (ease of change influencing behavior or vice versa) remains to be tested.

The reliance on self‐reported behaviors is also a limitation, given numerous accounts of overreporting of desirable and underreporting of undesirable behaviors (Paulhus, 1991; Stone, 2007). Nevertheless, the measures used are well validated and were selected based on their demonstrated validity. Still, the presented analyses should be replicated using objective measures, such as accelerometers for PA. Furthermore, the specific demographic and health profiles of the two study samples (young adults without chronic conditions vs. adults with chronic conditions) necessitate cautious interpretation of direct comparisons, as age and health status are inherently confounded variables influencing perceptions and behaviors. The demographic homogeneity of the samples with a predominance of White, highly educated individuals limits the generalizability of the findings, and further research using representative samples is required. Additionally, in Study 1, we did not controll for bots/scam responses, even though we do not expect the likelihood of such phenomena to be high as no incentives to take part in the study were offered (Kramer et al., 2014; Pozzar et al., 2020; Teitcher et al., 2015). In Study 2, bot control strategies were integrated by the data collection panel.

## CONCLUSION

These findings highlight the critical need for tailored, person‐centered interventions. Health promotion strategies should move beyond a one‐size‐fits‐all approach, recognizing that what constitutes an “easy” or “difficult” change varies significantly between individuals. For young adults, interventions might leverage their self‐efficacy for specific, measurable gains and integrate social components. For individuals with chronic conditions, interventions should prioritize manageable, health‐relevant changes, potentially focusing on reducing sedentary behavior or small, sustainable dietary shifts, while explicitly addressing potential barriers like pain and fatigue. Behavior change plans should ideally be co‐created with individuals, allowing their perceptions of ease and difficulty to guide goal setting and foster greater self‐regulatory processes (Hutchesson et al., 2015; Michie et al., 2011, 2009; Schau et al., 2025; Sniehotta, 2009).

### Supplementary material

The supplementary information presents further results of the statistical analyses for both studies.

## CONFLICT OF INTEREST STATEMENT

The authors declare no conflicts of interest.

## ETHICS STATEMENT

Study 1 was reviewed and approved by the ethics committee of the University of Bayreuth, Germany. Study 2 was reviewed and approved by the Institutional Review Board at Clemson University.

## Supporting information


**Table A:** Perceived ease of changing eating and physical activity behaviors. Means (M) and standard deviations (SD) for Studies 1 and 2.
Table B: Pairwise Comparison of Eating Behavior Aspects (Study 1).
Table C: Pairwise Comparison of Physical Activity Aspects (Study 1).
Table D: Pearson's Correlation Social Comparison‐Aspects of Eating Behavior (Study 1).
Table E: Pearson's Correlation Social Comparison‐Aspects of Physical Activity (Study 1).
Table F: Pearson's Correlation Previous attempts to change behavior‐Aspects of Eating Behavior (Study 1).
Table G: Pearson's Correlation Previous attempts to change behavior‐Aspects of Physical Activity (Study 1).
Table H: Pairwise Comparison of Eating Behavior Aspects (Study 2).
Table I: Pairwise Comparison of Physical Activity Aspects (Study 2).
Table J: Pearson's Correlation Social Comparison‐Aspects of Physical Activity (Study 2).
Table K: Pearson's Correlation Social Comparison‐Aspects of Eating Behavior (Study 2).
Table L: Pearson's Correlation Previous attempts to change behavior‐Aspects of Eating Behavior (Study 2).
Table M: Pearson's Correlation Previous attempts to change behavior‐Aspects of Physical Activity (Study 2).

## Data Availability

The datasets generated and analyzed during the current study are available in the OSF repository (https://osf.io/pukdg/).

## Footnotes

^1^
In the preregistration, this was mistakenly noted as N = 500

^2^
In the preregistration, this was mistakenly noted as aged 50 and older.
