# Supplementary material for: Easy or difficult? Investigating perceived ease of changing eating and physical activity behaviors
Source: Appl Psychol Health Well Being. 2026 Feb 23;18(1):e70124. doi: 10.1111/aphw.70124 (PMC12926927; doi:10.1111/aphw.70124)
Supplement: Supplementary file 1 — Table A: Perceived ease of changing eating and physical activity behaviors. Means (M) and standard deviations (SD) for Studies 1 and 2. Table B: Pairwise Comparison of Eating Behavior Aspects (Study 1). Table C: Pairwise Comparison of Physical Activity Aspects (Study 1). Table D: Pearson's Correlation Social Comparison‐Aspects of Eating Behavior (Study 1). Table E: Pearson's Correlation Social Comparison‐Aspects of Physical Activity (Study 1). Table F: Pearson's Correlation Previous attempts to change behavior‐Aspects of Eating Behavior (Study 1). Table G: Pearson's Correlation Previous attempts to change behavior‐Aspects of Physical Activity (Study 1). Table H: Pairwise Comparison of Eating Behavior Aspects (Study 2). Table I: Pairwise Comparison of Physical Activity Aspects (Study 2). Table J: Pearson's Correlation Social Comparison‐Aspects of Physical Activity (Study 2). Table K: Pearson's Correlation Social Comparison‐Aspects of Eating Behavior (Study 2). Table L: Pearson's Correlation Previous attempts to change behavior‐Aspects of Eating Behavior (Study 2). Table M: Pearson's Correlation Previous attempts to change behavior‐Aspects of Physical Activity (Study 2). [file APHW-18-0-s001.docx]

**Supplementary Materials Ease of Change Study 1 + 2**

**List:**

Table A: Perceived ease of changing eating and physical activity behaviours. Means (M) and standard deviations (SD) for Studies 1 and 2

Table B: Pairwise Comparison of Eating Behavior Aspects (Study 1)

Table C: Pairwise Comparison of Physical Activity Aspects (Study 1)

Table D: Pearson´s Correlation Social Comparison-Aspects of Eating Behavior (Study 1)

Table E: Pearson´s Correlation Social Comparison-Aspects of Physical Activity (Study 1)

Table F: Pearson´s Correlation Previous attempts to change behavior-Aspects of Eating Behavior (Study 1)

Table G: Pearson´s Correlation Previous attempts to change behavior-Aspects of Physical Activity (Study 1)

Table H: Pairwise Comparison of Eating Behavior Aspects (Study 2)

Table I: Pairwise Comparison of Physical Activity Aspects (Study 2)

Table J: Pearson´s Correlation Social Comparison-Aspects of Physical Activity (Study 2)

Table K: Pearson´s Correlation Social Comparison-Aspects of Eating Behavior (Study 2)

Table L: Pearson´s Correlation Previous attempts to change behavior-Aspects of Eating Behavior (Study 2)

Table M: Pearson´s Correlation Previous attempts to change behavior-Aspects of Physical Activity (Study 2)

| **Table A.**  *Perceived ease of changing eating and physical activity behaviours. Means (M) and standard deviations (SD) for Studies 1 and 2* | | | | |
| --- | --- | --- | --- | --- |
| **Aspects of Behavior** | **Study 1** (*N*=435) | | **Study 2** (*N*=496) | |
|  | **M** | ***SD*** | **M** | ***SD*** |
| **Eating Behavior:** |  |  |  |  |
| Eat no more than 300-600gr of meat/week | 3.68 | 1.54 | 3.51 | 1.58 |
| Eat healthier | 3.48 | 1.10 | 4.12 | 1.36 |
| Eat more snacks | 3.46 | 1.23 | 3.90 | 1.45 |
| Eat more overall | 3.33 | 1.29 | 3.06 | 1.26 |
| Eat 5 portions of fruit and vegetables | 3.30 | 1.46 | 3.60 | 1.57 |
| Eat additional meals | 3.18 | 1.34 | 3.49 | 1.51 |
| Eat larger portions | 3.09 | 1.34 | 3.54 | 1.52 |
| Eat fewer snacks | 3.06 | 1.26 | 3.87 | 1.43 |
| Eat no more than 50gr of added sugar/day | 3.06 | 1.36 | 3.83 | 1.48 |
| Eat less healthy | 3.03 | 1.35 | 3.78 | 1.47 |
| Eat smaller portions | 2.84 | 1.21 | 4.15 | 1.29 |
| Skip meals | 2.77 | 1.42 | 3.77 | 1.55 |
| Eat less overall | 2.72 | 1.20 | 3.93 | 1.32 |
| **Physical Activity Behaviour:** |  |  |  |  |
| Spend less time standing | 3.08 | 1.22 | 4.23 | 1.34 |
| Be more physically active overall | 3.00 | 1.19 | 3.79 | 1.54 |
| Spend more time sitting | 2.99 | 1.28 | 3.99 | 1.41 |
| Engage in moderate/vigorous physical activity for 300min/week | 2.97 | 1.35 | 3.07 | 1.68 |
| Walk at least 10.000 steps/day | 2.96 | 1.33 | 3.32 | 1.63 |
| Spend more time standing | 2.94 | 1.18 | 3.58 | 1.60 |
| Be less physically active overall | 2.79 | 1.38 | 3.72 | 1.51 |
| Spend less time sitting | 2.74 | 1.25 | 3.76 | 1.42 |
|  | | | | |

| **Table B.**  *Pairwise Comparison of Eating Behavior Aspects (Study 1)* | | | | | | | | | | | | |  |
| --- | --- | --- | --- | --- | --- | --- | --- | --- | --- | --- | --- | --- | --- |
| **Eating Behavior Aspects** | 2 | 3 | 4 | 5 | 6 | 7 | 8 | 9 | 10 | 11 | 12 | 13 |  |
| 1 | <.001* | .030* | <.001* | <.001* | <.001* | .515 | <.001* | <.001* | <.001* | <.001* | <.001* | <.001* |  |
| 2 | - | <.001* | <.001* | .055 | <.001* | <.001* | .017* | .002* | .036* | .802 | <.001* | .005* |  |
| 3 | - | - | .010* | <.001* | .042* | .356 | <.001* | .003* | <.001* | <.001* | <.001* | .006* |  |
| 4 | - | - | - | <.001* | .370 | <.001* | .167 | .739 | <.001* | .020* | <.001* | .773 |  |
| 5 | - | - | - | - | <.001* | <.001* | <.001* | <.001* | .797 | .011* | .017* | <.001* |  |
| 6 | - | - | - | - | - | .004* | .049* | .716 | <.001* | .008* | <.001* | .716 |  |
| 7 | - | - | - | - | - | - | <.001* | <.001* | <.001* | <.001* | <.001* | .001* |  |
| 8 | - | - | - | - | - | - | - | .188 | <.001* | .195 | <.001* | .220 |  |
| 9 | - | - | - | - | - | - | - | - | <.001* | .004* | <.001* | .975 |  |
| 10 | - | - | - | - | - | - | - | - | - | .076 | .019* | <.001* |  |
| 11 | - | - | - | - | - | - | - | - | - | - | <.001* | .004* |  |
| 12 | - | - | - | -- | - | - | - | - | - | - | - | <.001* |  |
| *Note***. Eating related aspects**: 1= Eat less overall; 2= Eat more overall; 3= Eat smaller portions; 4= Eat larger portions; 5= Eat healthier; 6= Eat less healthy; 7= Skip meals; 8= Eat an additional meal; 9= Eat fewer snacks; 10= Eat more snacks; 11= Eat five portions of fruits and vegetables per day; 12= Eat no more than 300 to 600 grams of meat per week; 13= Eat no more than 50 grams of added sugar per day  *Correlation is significant at the 0.05 level (2-tailed) | | | | | | | | | | | | | |

| **Table C.**  *Pairwise Comparison of Physical Activity Aspects (Study 1)* | | | | | | | |
| --- | --- | --- | --- | --- | --- | --- | --- |
| **Physical Activity Aspects** | 2 | 3 | 4 | 5 | 6 | 7 | 8 |
| 1 | .048* | .471 | .351 | <.001* | .980 | .582 | .690 |
| 2 | - | .096 | <.001* | .599 | .005* | .107 | .096 |
| 3 | - | - | .165 | .001* | .529 | .870 | .758 |
| 4 | - | - | - | <.001* | .180 | .193 | .257 |
| 5 | - | - | - | - | .010* | .003* | .002* |
| 6 | - | - | - | - | - | .698 | .793 |
| 7 | - | - | - | - | - | - | .861 |
| *Note.* **Physical Activity aspects**: 1= Be more physically active 2= Be less physically active; 3= Spend more time standing; 4= Spend less time standing; 5= Spend less time sitting; 6= Spend more time sitting; 7= Walk at least 10000 steps per day; 8= Engage in moderate to high intensity activities for at least 300 minutes per week  *Correlation is significant at the 0.05 level (2-tailed) | | | | | | | |

**Correlations Tables Study 1**

**Study 1** (Social Comparison; Previous attempt to change eating behavior and PA)

| **Table D.**  *Pearson´s Correlation Social Comparison-Aspects of Eating Behavior (Study 1)* | | | | |
| --- | --- | --- | --- | --- |
| **Aspects of Eating Behavior** | **Social Comparison** | | | |
|  | How healthy are their eating behaviors in comparison to others | | How often they compare own eating behaviors to others | |
|  | ***r*** | ***p*** | ***r*** | ***p*** |
| Eat Less Overall | .086 | .075 | -.019 | .699 |
| Eat more overall | .102 | .034* | -.068 | .157 |
| Eat smaller portions | -.019 | .698 | .101 | .036* |
| Eat larger portions | -.048 | .318 | .121 | .012* |
| Eat healthier | .395 | <.001* | .083 | .085 |
| Eat less healthy | -.330 | .<.001* | .028 | .560 |
| Skip meals | -.034 | .484 | -.012 | .804 |
| Eat additional meals | -.037 | .437 | .090 | .059 |
| Eat fewer snacks | .203 | <.001* | -.035 | .472 |
| Eat more snacks | -.085 | .076 | .074 | .123 |
| Eat 5 portions of fruit and vegetables | .339 | <.001* | .012 | .806 |
| Eat no more than 300-600gr of meat/week | .141 | .003* | .018 | .709 |
| Eat no more than 50gr of added sugar/day | .301 | <.001* | .024 | .611 |
|  | **N**=435 | | **N**=435 | |
| *Note.* N= number of participants in the analysis  *Correlation is significant at the 0.05 level (2-tailed) | | | | |

| **Table E.**  *Pearson´s Correlation Social Comparison-Aspects of Physical Activity (Study 1)* | | | | |
| --- | --- | --- | --- | --- |
| **Aspects of Physical Activity** | **Social Comparison** | | | |
|  | How PA they think they are in comparison to others | | How often they compare own PA levels to others | |
|  | ***r*** | ***p*** | ***r*** | ***p*** |
| Be more physically active | .066 | .172 | .339 | <.001* |
| Be less physically active | -.039 | .413 | -.374 | <.001* |
| Spend more time standing | .093 | .053 | .143 | .003* |
| Spend less time standing | .002 | .973 | -.144 | .003* |
| Spend less time sitting | .055 | .255 | .226 | <.001* |
| Spend more time sitting | -.022 | .641 | -.256 | <.001* |
| Walk at least 10000 steps per day | .093 | .053 | .343 | <.001* |
| Engage in moderate to high intensity activities for at least 300 minutes per week | .126 | .008* | .598 | <.001* |
|  | **N**=435 | | **N**=435 | |
| *Note*. N= number of participants in the analysis  *Correlation is significant at the 0.05 level (2-tailed) | | | | |

| **Table F.**  *Pearson´s Correlation Previous attempts to change behavior-Aspects of Eating Behavior (Study 1)* | | | | | | | | |
| --- | --- | --- | --- | --- | --- | --- | --- | --- |
| **Aspects of Eating Behavior** | **Previous Attempts to change Eating Behavior** | | | | | | | |
|  | Improve Eating Behavior | | Start a formal healthy eating program | | Start healthy eating program (< 3 days) | | Start healthy eating program (≥ 3 days) | |
|  | ***r*** | ***p*** | ***r*** | ***p*** | ***r*** | ***p*** | ***r*** | ***p*** |
| Eat Less Overall | -.053 | .283 | .070 | .155 | .055 | .268 | .025 | .608 |
| Eat more overall | .154 | .002* | .014 | .781 | .044 | .375 | .026 | .602 |
| Eat smaller portions | -.072 | .147 | .054 | .275 | -.058 | .238 | -.053 | .288 |
| Eat larger portions | .114 | .021* | -.038 | .437 | .014 | .776 | .011 | .831 |
| Eat healthier | -.034 | .487 | .058 | .233 | .041 | .412 | .024 | .634 |
| Eat less healthy | .131 | .008* | -.041 | .408 | .020 | .690 | .038 | .442 |
| Skip meals | .009 | .864 | .078 | .113 | .006 | .907 | .089 | .071 |
| Eat additional meals | .105 | .034* | .109 | .025* | -.002 | .968 | .065 | .188 |
| Eat fewer snacks | -.040 | .425 | .059 | .229 | -.092 | .061 | -.019 | .700 |
| Eat more snacks | .063 | .203 | .089 | .070 | .019 | .698 | -.022 | .652 |
| Eat 5 portions of fruit and vegetables | -.086 | .084 | .003 | .959 | .047 | .338 | -.021 | .674 |
| Eat no more than 300-600gr of meat/week | -.043 | .385 | -.092 | .059 | -.085 | .084 | -.009 | .853 |
| Eat no more than 50gr of added sugar/day | -.042 | .396 | -.083 | .090 | .014 | .771 | .014 | .781 |
|  | **N**=435 | | **N**=435 | | **N**=435 | | **N**=435 | |
| *Note*. N= number of participants in the analysis  *Correlation is significant at the 0.05 level (2-tailed) | | | | | | | | |

| **Table G.**  *Pearson´s Correlation Previous attempts to change behavior-Aspects of Physical Activity (Study 1)* | | | | | | | | |
| --- | --- | --- | --- | --- | --- | --- | --- | --- |
| **Aspects of Physical Activity** | **Previous Attempts to change Physical Activity Levels** | | | | | | | |
|  | Improve PA Level | | Start a formal PA program | | Start PA program (< 3 days) | | Start PA program (≥ 3 days) | |
|  | ***r*** | ***p*** | ***r*** | ***p*** | ***r*** | ***p*** | ***r*** | ***p*** |
| Be more physically active | .059 | .243 | .066 | .183 | -.059 | .235 | .019 | .703 |
| Be less physically active | .106 | .036* | -.039 | .430 | -.059 | .230 | -.015 | .762 |
| Spend more time standing | -.005 | .925 | .037 | .457 | .079 | .111 | .024 | .627 |
| Spend less time standing | -.057 | .245 | -.044 | .369 | .005 | .915 | .025 | .617 |
| Spend less time sitting | .067 | .177 | .043 | .382 | -.010 | .841 | -.042 | .398 |
| Spend more time sitting | -.027 | .591 | -.006 | .907 | .078 | .113 | .084 | .091 |
| Walk at least 10000 steps per day | -.077 | .120 | .097 | .049* | .007 | .891 | .005 | .915 |
| Engage in moderate to high intensity activities for at least 300 minutes per week | .073 | .139 | .071 | .151 | .057 | .253 | .050 | .316 |
|  | **N**=435 | | **N**=435 | | **N**=435 | | **N**=435 | |
| *Note*. PA = Physical Activity, N= number of participants in the analysis  *Correlation is significant at the 0.05 level (2-tailed) | | | | | | | | |

Study 2:

| **Table H.**  *Pairwise Comparison of Eating Behavior Aspects (Study 2)* | | | | | | | | | | | | |  |
| --- | --- | --- | --- | --- | --- | --- | --- | --- | --- | --- | --- | --- | --- |
| **Eating Behavior Aspects** | 2 | 3 | 4 | 5 | 6 | 7 | 8 | 9 | 10 | 11 | 12 | 13 |  |
| 1 | .101 | <.001* | <.001* | .004* | .084 | .025* | <.001* | .315 | .711 | <.001* | <.001* | .162 |  |
| 2 | - | <.001* | <.001* | <.001* | .979 | .916 | <.001* | .328 | .078 | .040* | .007* | .589 |  |
| 3 | - | - | <.001* | .587 | <.001* | <.001* | <.001* | <.001* | .006* | <.001* | <.001* | <.001* |  |
| 4 | - | - | - | <.001* | .002* | .024* | .425 | <.001* | <.001* | .527 | .763 | .003* |  |
| 5 | - | - | - | - | <.001* | <.001* | <.001* | <.001* | .018* | <.001* | <.001* | <.001* |  |
| 6 | - | - | - | - | - | .885 | <.001* | .363 | .094 | .071 | .009* | .633 |  |
| 7 | - | - | - | - | - | - | .003* | .235 | .160 | .079 | .006* | .510 |  |
| 8 | - | - | - | - | - | - | - | <.001* | <.001* | .230 | .811 | <.001* |  |
| 9 | - | - | - | - | - | - | - | - | .758 | .001* | <.001* | .603 |  |
| 10 | - | - | - | - | - | - | - | - | - | .001* | <.001* | .451 |  |
| 11 | - | - | - | - | - | - | - | - | - | - | .307 | .004* |  |
| 12 | - | - | - | -- | - | - | - | - | - | - | - | <.001* |  |
| *Note***. Eating related aspects**: 1= Eat less overall; 2= Eat more overall; 3= Eat smaller portions; 4= Eat larger portions; 5= Eat healthier; 6= Eat less healthy; 7= Skip meals; 8= Eat an additional meal; 9= Eat fewer snacks; 10= Eat more snacks; 11= Eat five portions of fruits and vegetables per day; 12= Eat no more than 300 to 600 grams of meat per week; 13= Eat no more than 50 grams of added sugar per day  *Correlation is significant at the 0.05 level (2-tailed) | | | | | | | | | | | | | |

| **Table I.**  *Pairwise Comparison of Physical Activity Aspects (Study 2)* | | | | | | | |
| --- | --- | --- | --- | --- | --- | --- | --- |
| **Physical Activity Aspects** | 2 | 3 | 4 | 5 | 6 | 7 | 8 |
| 1 | .522 | .057 | <.001* | .567 | .005* | <.001* | <.001* |
| 2 | - | .052 | <.001* | .741 | .005* | <.001* | <.001* |
| 3 | - | - | <.001* | .005* | <.001* | <.001* | <.001* |
| 4 | - | - | - | <.001* | <.001* | <.001* | <.001* |
| 5 | - | - | - | - | .014* | <.001* | <.001* |
| 6 | - | - | - | - | - | <.001* | <.001* |
| 7 | - | - | - | - | - | - | <.001* |
| *Note***. Physical Activity aspects**: 1= Be more physically active 2= Be less physically active; 3= Spend more time standing; 4= Spend less time standing; 5= Spend less time sitting; 6= Spend more time sitting; 7= Walk at least 10000 steps per day; 8= Engage in moderate to high intensity activities for at least 300 minutes per week  *Correlation is significant at the 0.05 level (2-tailed) | | | | | | | |

**Correlations Tables Study 2**

**Study 2** (Social Comparison; Previous attempt to change eating behavior and PA)

| **Table J.**  *Pearson´s Correlation Social Comparison-Aspects of Physical Activity (Study 2)* | | | | |
| --- | --- | --- | --- | --- |
| **Aspects of Physical Activity** | **Social Comparison** | | | |
|  | How PA they think they are in comparison to others | | How often they compare own PA levels to others | |
|  | ***r*** | ***p*** | ***r*** | ***p*** |
| Be more physically active | .54 | <.001* | .04 | .389 |
| Be less physically active | -.28 | <.001* | .01 | .826 |
| Spend more time standing | .45 | <.001* | .06 | .211 |
| Spend less time standing | -.17 | <.001* | .02 | .603 |
| Spend less time sitting | .39 | <.001* | .01 | .783 |
| Spend more time sitting | -.18 | <.001* | .05 | .221 |
| Walk at least 10000 steps per day | .55 | <.001* | .07 | .098 |
| Engage in moderate to high intensity activities for at least 300 minutes per week | .59 | <.001* | .12 | .007* |
|  | **N**=507 | | **N**=507 | |
| *Note*. N= number of participants in the analysis  *Correlation is significant at the 0.05 level (2-tailed) | | | | |

| **Table K.**  *Pearson´s Correlation Social Comparison-Aspects of Eating Behavior (Study 2)* | | | | |
| --- | --- | --- | --- | --- |
| **Aspects of Eating Behavior** | **Social Comparison** | | | |
|  | How healthy are their eating behaviors in comparison to others | | How often they compare own eating behaviors to others | |
|  | ***r*** | ***p*** | ***r*** | ***p*** |
| Eat Less Overall | .28 | <.001* | -.06 | .145 |
| Eat more overall | -.02 | .682 | .08 | .065 |
| Eat smaller portions | .25 | <.001* | .002 | .968 |
| Eat larger portions | -.07 | .103 | .08 | .081 |
| Eat healthier | .48 | <.001* | .04 | .343 |
| Eat less healthy | -.26 | .<.001* | -.01 | .841 |
| Skip meals | -.03 | .488 | -.05 | .276 |
| Eat additional meals | -.06 | .203 | .08 | .088 |
| Eat fewer snacks | .28 | <.001* | .05 | .911 |
| Eat more snacks | -.08 | .057 | .02 | .700 |
| Eat 5 portions of fruit and vegetables | .29 | <.001* | .06 | .193 |
| Eat no more than 300-600gr of meat/week | .20 | <.001* | .06 | .182 |
| Eat no more than 50gr of added sugar/day | .27 | <.001* | .01 | .758 |
|  | **N**=510 | | **N**=510 | |
| *Note*. N= number of participants in the analysis  *Correlation is significant at the 0.05 level (2-tailed) | | | | |

| **Table L.**  *Pearson´s Correlation Previous attempts to change behavior-Aspects of Eating Behavior (Study 2)* | | | | | | |
| --- | --- | --- | --- | --- | --- | --- |
| **Aspects of Eating Behavior** | **Previous Attempts to change Eating Behavior** | | | | | |
|  | Start a formal healthy eating program | | Start healthy eating program (< 3 days) | | Start healthy eating program (≥ 3 days) | |
|  | ***r*** | ***p*** | ***r*** | ***p*** | ***r*** | ***p*** |
| Eat Less Overall | .12 | .007* | .08 | .083 | .09 | .039* |
| Eat more overall | .03 | .938 | -.03 | .459 | -05 | .215 |
| Eat smaller portions | .08 | .068 | .06 | .141 | .06 | .145 |
| Eat larger portions | .02 | .596 | -.02 | .740 | -.04 | .427 |
| Eat healthier | .13 | .003* | .11 | .010* | .10 | .031* |
| Eat less healthy | .02 | .720 | -.05 | .257 | -.04 | .393 |
| Skip meals | .04 | .371 | .02 | .721 | .01 | .748 |
| Eat additional meals | .01 | .928 | -.05 | .281 | -.07 | .100 |
| Eat fewer snacks | .07 | .113 | .05 | .276 | .05 | .279 |
| Eat more snacks | -.10 | .022* | -.07 | .109 | -.09 | .050 |
| Eat 5 portions of fruit and vegetables | .03 | .499 | .03 | .522 | .01 | .838 |
| Eat no more than 300-600gr of meat/week | .04 | .402 | .01 | .787 | .02 | .711 |
| Eat no more than 50gr of added sugar/day | .10 | .030* | .09 | .051 | .11 | .015* |
|  | **N**=507 | | **N**=507 | | **N**=507 | |
| *Note*. N= number of participants in the analysis  *Correlation is significant at the 0.05 level (2-tailed) | | | | | | |

| **Table M.**  *Pearson´s Correlation Previous attempts to change behavior-Aspects of Physical Activity (Study 2)* | | | | | | | | |
| --- | --- | --- | --- | --- | --- | --- | --- | --- |
| **Aspects of Physical Activity** | **Previous Attempts to change Physical Activity Levels** | | | | | | | |
|  | Improve PA Level | | Start a formal PA program | | Start PA program (< 3 days) | | Start PA program (≥ 3 days) | |
|  | ***r*** | ***p*** | ***r*** | ***p*** | ***r*** | ***p*** | ***r*** | ***p*** |
| Be more physically active | -.06 | .186 | .05 | .280 | -.02 | .724 | .01 | .889 |
| Be less physically active | -.05 | .305 | -.06 | .172 | -.10 | .024* | -.06 | .194 |
| Spend more time standing | -.05 | .238 | .04 | .356 | -02 | .629 | -.04 | .391 |
| Spend less time standing | .03 | .476 | .01 | .901 | -.06 | .182 | -.03 | .473 |
| Spend less time sitting | -.02 | .702 | .03 | .492 | .01 | .821 | .01 | .878 |
| Spend more time sitting | -.03 | .469 | .03 | .487 | -.06 | .188 | -.02 | .689 |
| Walk at least 10000 steps per day | -.02 | .710 | .05 | .261 | .08 | .058 | .03 | .480 |
| Engage in moderate to high intensity activities for at least 300 minutes per week | -.01 | .921 | .09 | .051 | .10 | .020* | -.01 | .970 |
|  | **N**=507 | | **N**=507 | | **N**=507 | | **N**=507 | |
| *Note*. N= number of participants in the analysis  *Correlation is significant at the 0.05 level (2-tailed) | | | | | | | | |
